# Unconventional Therapy with IgY in a Psoriatic Mouse Model Targeting Gut Microbiome

**DOI:** 10.3390/jpm11090841

**Published:** 2021-08-26

**Authors:** Mihaela Surcel, Adriana Munteanu, Gheorghita Isvoranu, Alef Ibram, Constantin Caruntu, Carolina Constantin, Monica Neagu

**Affiliations:** 1 Immunology Department, “Victor Babes” National Institute of Pathology, 99-101 Spl. Independentei, 050096 Bucharest, Romania; msurcel2002@yahoo.com (M.S.); iadinuta@yahoo.com (A.M.); caroconstantin@gmail.com (C.C.); 2 Faculty of Biology, Doctoral School of Biology, University of Bucharest, 91-95 Spl. Independentei, 030018 Bucharest, Romania; 3 Animal Husbandry, “Victor Babes” National Institute of Pathology, 99-101 Spl. Independentei, 050096 Bucharest, Romania; gina_isvoranu@yahoo.com; 4 Research Laboratory, Romvac Company SA, 077190 Voluntari, Romania; mustafa_alef@yahoo.com; 5 Department of Physiology, “Carol Davila” University of Pharmacy and Medicine, 37 Dionisie Lupu Street, 020021 Bucharest, Romania; costin.caruntu@gmail.com; 6 Department of Pathology, Colentina University Hospital, 020125 Bucharest, Romania

**Keywords:** IgY, psoriatic dermatitis, imiquimod, inflammation, C57 BL/6 mice

## Abstract

Psoriasis has a multifactorial pathogenesis and recently it was shown that alterations in the skin and intestinal microbiome are involved in the pathogenesis of psoriasis. Therefore, microbiome restoration becomes a promising preventive/therapy strategy in psoriasis. In our pre-clinical study design using a mice model of induced psoriatic dermatitis (Ps) we have tested the proof-of-concept that IgY raised against pathological human bacteria resistant to antibiotics can alleviate psoriatic lesions and restore deregulated immune cell parameters. Besides clinical evaluation of the mice and histology of the developed psoriatic lesions, cellular immune parameters were monitored. Immune cells populations/subpopulations from peripheral blood and spleen cell suspensions that follow the clinical improvement were assessed using flow cytometry. We have quantified T lymphocytes (CD3ε^+^) with T-helper (CD4^+^CD8^−^) and T-suppressor/cytotoxic (CD8a^+^CD4^−^) subsets, B lymphocytes (CD3ε^−^CD19^+^) and NK cells (CD3ε^−^NK1.1^+^). Improved clinical evolution of the induced Ps along with the restoration of immune cells parameters were obtained when orally IgY was administered. We pin-point that IgY specific compound can be used as a possible pre-biotic-like alternative adjuvant in psoriasis.

## 1. Introduction

Psoriasis (Ps) is a complex and heterogeneous disease that affects not only the skin of the patients but echoes also on several other organs [1]. Recognized as a chronic autoimmune inflammatory disease, mediated mainly by T cells, Ps has important systemic manifestation [2], being frequently associated with psychological, metabolic, arthritic and cardiovascular comorbidities. Ps associated pathologies can lead to increased mortality and alters the clinical management of the patients. Ps affects 0.5–1% of children and in the world’s population the prevalence raises around 2–3%. Prevalence of Ps varies depending on age, sex, geography, ethnicity, genetic and environmental factors [3]. Although it can occur at any age, the most common cases are reported before the age of 35, an age range that affects highly active individuals [4].

The causes of Ps have not yet been fully elucidated. Besides genetic predisposition and environmental factors, an inefficient immune system is highly involved in Ps onset. Among the triggers or aggravating factors of Ps we can mention air pollutants and exposure to sunlight, prolonged exposure to UV radiation [5], administration of certain drugs (β-blockers, lithium) [6], smoking [7], obesity [8] and alcohol consumption [9]. Streptococcal infections, involved in both acute and chronic forms of the disease [10] and mental stress are also factors that have with critical roles in the initiation, development and exacerbation of Ps [11].

The assessment of Ps severity is mainly based on clinical indicators [12], but the golden standard remains the PASI score (Psoriasis Area Severity Index) [13], which combines the severity (erythema, scaling, induration) and the percentage of affected area.

19In Ps pathogenesis are involved both innate (NK cells, macrophages, dendritic cells) and adaptive immune cells (T lymphocytes), as well as non-immune cells (keratinocytes), their interactions being mediated by pro-inflammatory cytokines/chemokines that maintain the chronic inflammatory state [14,15]. Among the biological therapies currently available, TNF-α inhibitors [16,17,18], IL-23 inhibitors [19,20,21,22], IL-17 inhibitors [23,24,25] are the ones that entered the standard care of Ps. New treatments that address immune pathways and are currently undergoing clinical trials include RORγt inhibitors [26], IL-36 Receptor antagonist [27], Janus Kinase (JAK) inhibitors [28], TYK2/JAK1 inhibitor [29], Rho-Associated Kinase (ROCK2) inhibitor [30], Sphingosine-1-Phosphate (S1P) agonist [31] and aryl hydrocarbon receptor (AhR) agonists [32]. All current therapies display adverse effects, such as nasopharyngitis, upper respiratory tract infections, fatigue, headache and even tuberculosis, therefore adjuvant therapies that can aid the standard ones are searched.

Immunoglobulin Y (IgY) has important characteristics like high tolerability, being essentially a component of the human diet. Hence, it can be used even in subjects that are allergic to egg components because the purified IgY does not contain allergenic ovalbumin [33]. Since IgY cannot link to the Fc receptors or to the complement system expressed by the mammalian cells, administering IgY does not trigger adverse effects [34,35]. A report published more than 20 years ago has shown in animal models that purified IgY does not trigger an IgE response, therefore no allergic reaction [36]. Moreover, the systemic administration of IgY has shown that this Ig can have anti-viral and/or anti-bacterial potency [37]. IgY can neutralize bacteria and viruses, hindering their replication [38,39] therefore passive immunization can be used in humans because it will rapidly give a positive clinical response. In the last decade, IgY has gained an increased scientific attention due to its specific characteristic and biological potency [40]. 

In Ps, association of bacterial strains residing in the digestive tract of patients was reported, staring from the incidence of *H. pylori* [41], of *Candida albicans* [42] and ending with the major gut dysbiosis registered in these patients, dysbiosis that can influence the skin microbiota favoring thus flare-up of the psoriatic events [43].

Our previously published work has shown that in psoriatic dermatitis murine models, although involving just a psoriatic-like skin lesion, significant alterations of lymphocytes percentages and important changes in NK cell phenotype, in both peripheral blood and spleen were found [43,44]. Considering all the accumulated data, we have initiated in the current study a Ps experimental model in which an adjuvant therapy using oral IgY developed against several pathogenic bacteria to evaluate the potency to alleviate the psoriatic lesions and to restore the immune-related mechanisms. Imiquimod murine model (IMQ-1-isobutyl-1H-imidazo[4,5-c]quinolin-4-amine) was used to develop the experimental psoriatic dermatitis and further evaluate if the orally given IgY treatment would clinically improve the experimental Ps and would restore the cellular immune parameters.

## 2. Materials and Methods

### 2.1. Immunoglobulin Y

IgY was isolated from the yolk of hyperimmune eggs laid by chickens immunized with human pathogenic bacteria that are antibiotic-resistant, namely groups of pathogens with a high rate of antibiotic resistance responsible for the majority of nosocomial infections. IgY was obtained according to the methodology described in the patent [45]. The obtained IgY is an original product of the ROMVAC Company and is part of the IMUNOINSTANT brand having a European trademark (EUIPO). IgY that was used is a mix of antibodies raised against the following antibiotic-resistant strains Salmonella spp (enteritidis, typhimurium), Streptococcus pneumoniae, Clostridium difficile, Staphylococcus aureus, Pseudomonas aeruginosa, Enterococcus faecalis, Klebsiella pneumoniae, Acinetobacter baumannii, Escherichia coli [46].

### 2.2. Animal Model

C57 BL/6 mice (Jackson Laboratory, Bar Harbor, ME), males and females, aged 10–11 weeks, were provided by the Animal Husbandry from Victor Babeș National Institute of Pathology. The animals were kept in an open cage system, in optimal conditions (temperature 22 ± 2 °C, humidity 55 ± 10%, artificial ventilation, 12/12-light/dark cycle) and fed (standard granulated fodder) and watered (filtered and sterilized water) *ad libitum*. The mice were monitored daily. The experiments were conducted in accordance with recognized principles of laboratory animal care in the framework of EU Directive 2010/63/EU [45] and the study was comprised in a research project that was approved by the Ethics Committee from Victor Babeș Institute (Approval no 88/20 January 2021) and National Sanitary Veterinary and Food Safety Authority (Approval no 598/8 February 2021).

The experimental murine model of psoriatic dermatitis was performed according to the protocols previously described [43,44,47].

Four groups of C57 BL/6 mice, were constituted as follows:

- Ps group (8 mice-1:1 sex ratio, with a mean weight 20.4 ± 2.9 g) received a daily topical dose of 62.5 mg IMQ-based cream (5% Aldara Cream, Meda AB Sweden) on the shaved back region, for 6 consecutive days. The daily dose contains 3.125 mg of active compound. The mice that were designed for clinical and immunological evaluation were sacrificed on day 7 of the experiment;
- IgY-treated Ps group (12 mice-1:1 sex ratio, with a mean weight 21.33 ± 2.14 g) with induced psoriatic dermatitis as described above, received (starting with day 7) a gavage dose of 37.5 µg IgY, for 5 consecutive days; the dose matches the dose of IgY given to a human adult (g/kg) according to a study case [48]. Mice were sacrificed on day 20, the day on which it was macroscopically assessed that experimental psoriatic dermatitis was remitted;
- Naturally remitted Ps group (8 mice—1:1 sex ratio, with a mean weight 17.87 ± 0.81 g) with induced psoriatic dermatitis were allowed to heal naturally and were sacrificed on day 22—the day on which the natural remission was assessed macroscopically;
- Control group (8 mice—1:1 sex ratio, with a mean weight 20.26 ± 1.36 g). Healthy mice with no treatment housed and fed in the same room with all the presented experimental groups and subjected to the same manipulation as the IgY-treated group but with sham gavage.

### 2.3. Scoring Severity of IMQ-Induced Skin Inflammation and Healing Assesment

The severity of IMQ-induced skin inflammation and the progress of the disease, were daily evaluated using three in vivo parameters-erythema, thickening and skin scaling. These parameters were scored daily on a 0–4 scale such as 0—no change, 1—mild change, 2—marked change; 3—significant change, 4—severe change. By summing erythema, thickening and skin scaling daily scores, a modified PASI score was calculated (0–12 scale).

The body weight of the animals was monitored during the experiments (Scientech SL 3100D, Boulder, CO, USA). For the mice from IgY-treated Ps group the body weight was recorded at the beginning of the experiment (day 1), day 7 (beginning of IgY treatment), at the end of IgY treatment (day 12) and before being humanely euthanized—day 20—when it was macroscopically appreciated that the induced psoriasis disappeared. The mice from naturally remitted Ps group were allowed to heal naturally and weighed at the beginning of the experiment (day 1), after IMQ-based cream application (day 7) and before euthanization—day 22—when it was macroscopically appreciated that the effects produced by IMQ-based cream disappeared. The mice from Ps group were weighed at the beginning of the experiment (day 1) and before sacrifice (day 7).

### 2.4. Sampling of Biological Material and Processing of Samples

At the end of experiments, the animals were anesthetized with ketamine/acepromazine/xylazine cocktail (ketamine 80 mg/kg, Richterpharma ag, Wells, Austria; acepromazine 6 mg/kg, Vetoquinol SA, Lure, France; xylazine 1 mg/kg, Bioveta SA, Czech Republic) for blood, spleen and skin sample collection. Peripheral blood was collected by intra-cardiac puncture in K2-EDTA coated tubes (SARSTEDT AG & CO. KG, Nümbrecht, Germany). Spleens were weighed (Balance AEP-1500A, Adam Equipment Co., Ltd., Kingston, UK) for splenomegaly evaluation and processed in order to isolate the spleen cells. The spleens were collected in 5% FBS RPMI 1640 media (Biochrom AG GmbH, Berlin, Germany), passed through a 70 µm cell strainer (BD Falcon -BD Biosciences, San Jose, CA, USA), cells centrifuged for 5 min at 350× *g* (20 °C) and resuspended in RBC Lysis Buffer (BioLegend, San Diego, CA, USA). After 5 min on ice, 10 mL Cell Staining Buffer (BioLegend, San Diego, CA, USA) was added in order to stop the lysis and centrifuged for 5 min at 350× *g* (20 °C). The pellet was resuspended twice in Cell Staining Buffer, for a final concentration of 1 × 10^6^ cells/mL. Skin samples were collected and processed (fixed in 10% buffered formalin, embedded in paraffin, sectioned in 5 µm sections) for hematoxylin and eosin (H&E) staining, prior to histopathological evaluation (Olympus BX43 with CellSens Dimension Program, Tokyo, Japan).

### 2.5. Flow Cytometry Analysis

Lymphocyte immunophenotyping performed from peripheral blood and spleen cell suspension were done for all experimental groups by flow cytometry, using a BD FACSCanto II cytometer (BD Biosciences, San Jose, CA, USA). We have quantified T lymphocytes (CD3ε^+^), with T helper (CD4^+^CD8^−^) and T suppressor/cytotoxic (CD8a^+^CD4^−^) subsets, B lymphocytes (CD3ε^−^CD19^+^), NK cells (CD3ε^−^NK1.1^+^), and the expression levels of several maturation markers (CD49b, CD27, CD11b, CD43, KLRG1) and activation (CD69, CD28, CD11c, NKp46) markers on NK cells were assessed.

Both types of samples (peripheral blood and spleen cell suspension) were incubated with TruStain fcX (anti-mouse CD16/32, isotype Rat IgG2a, λ) Antibody (BioLegend, San Diego, CA, USA) for 7 min on ice and stained in the dark for 20 min at room temperature with the following monoclonal antibodies conjugated with fluorochromes: 0.5 µL Alexa Fluor 647 anti-mouse CD3ε (clone 145-2C11, isotype Armenian Hamster IgG); 0.5 µL Alexa Fluor 488 anti-mouse CD8a (clone 53–6.7, isotype Rat IgG2a, κ); 1.25 µL PE-Cy7 anti-mouse CD4 (clone GK1.5, isotype Rat IgG2b, κ); 1,25 µL PerCP-Cy5.5 anti-mouse CD19 (clone 6D5, isotype Rat IgG2a, κ); 1,25 µL PE anti-mouse NK1.1 (clone PK136, isotype Mouse IgG2a, κ); 0.5 µL FITC anti-mouse CD3ε (clone 145-2C11, isotype Armenian Hamster IgG); 2.5 µL Brilliant Violet 510 anti-mouse NK1.1 (clone PK136, isotype Mouse IgG2a, κ); 0.6 µL PerCP/Cy5.5 anti-mouse/rat/human CD27 (clone LG.3A10, isotype Armenian Hamster IgG); 0.6 µL APC/Cy7 anti-mouse CD43 (clone RA3-6B2, isotype Rat IgG2a, κ); 1.25 µL PE anti-mouse CD28 (clone 37.51, isotype Syrian Hamster IgG); 2.5 µL PE/Cy7 anti-mouse CD69 (clone H1.2F3, isotype Armenian Hamster IgG); 2.5 µL PE/Cy7 anti-mouse CD335 (NKp46) (clone 29A1.4, isotype Rat IgG2a, κ); 2.5 µL PerCP/Cy5.5 anti-mouse CD11c (clone N418, isotype Armenian Hamster IgG) (all from BioLegend, San Diego, CA, USA); 2.5 µL eFluor 450 anti-mouse CD49b (clone DX5, isotype Rat IgM, κ); 0.3 µL APC anti-mouse CD11b (clone M1/70, isotype Rat IgG2b, κ); 0.6 µL PE anti-mouse KLRG1 (clone 2F1, isotype Syrian Hamster IgG) (all from eBioscience Inc, San Diego, CA, USA). Red blood cells lysis was performed with BD FACS Lysing Solution (BD Biosciences, San Jose, CA, USA) for 10 min in the dark at room temperature, followed by centrifugation for 5 min at 350× *g* and two washing steps with Cell Staining Buffer. Flow cytometry analysis was preceded by daily check-up of cytometer performances (BD Cytometer Setup and Tracking Beads Kit, BD Biosciences, San Jose, CA, USA) and compensation of spectral overlaps (UltraComp eBeads, Invitrogen by Thermo Fischer Scientific, San Diego, CA, USA). Unlabeled cells were used as negative control. Data were acquired and analyzed using BD FACSDiva v 6.1 software (BD Biosciences, San Jose, CA, USA).

### 2.6. Statistical Analysis

The results were expressed as mean values ± SD, and Microsoft Excel (Microsoft, Redmond, CA, USA) was used for data analysis. Student’s *t*-test (two-tailed, assuming equal variance) was used to compare the experimental groups, and a *p*-value less than 0.05 was considered statistically significant. T-CD4^+^ and T-CD8a^+^ were expressed as percentages of CD3ε^+^ lymphocytes (mean values ± SD), B and NK cells as percentages of CD3ε^−^ lymphocytes (mean values ± SD), and the expressions of maturation and activation markers on NK cells, as percentages of NK1.1^+^ cells gated from CD3ε^−^ lymphocytes (mean values ± SD).

## 3. Results

### 3.1. IMQ-Based Experimental Murine Model of Psoriatic Dermatitis

**Figure 1:**
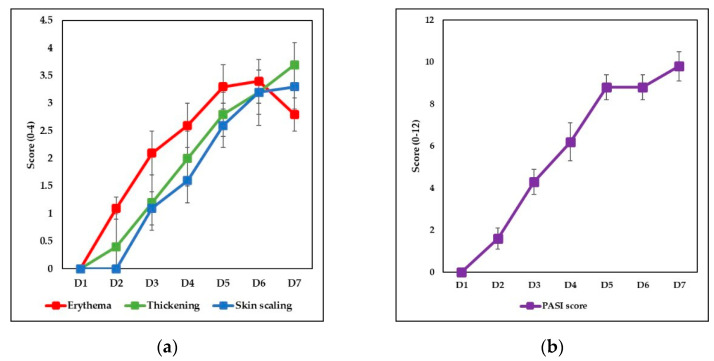

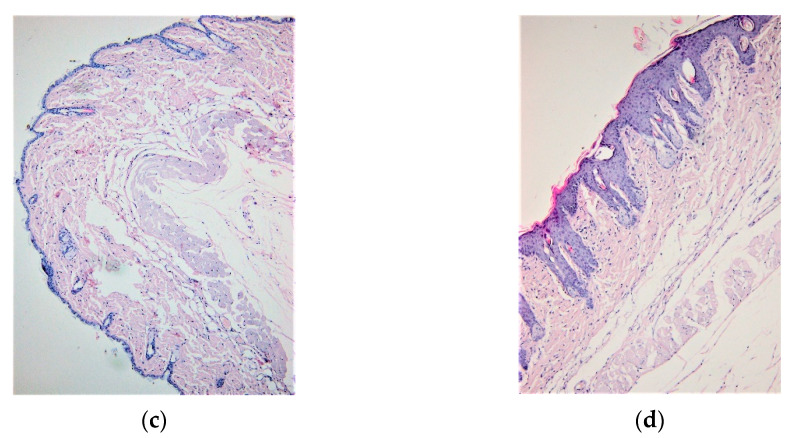
Evolution of in vivo parameters and histopathological assessment of dorsal skin samples. (**a**) In vivo measurements scores for erythema (0 ± 0; 1.1 ± 0.2; 2.1 ± 0.4; 2.6 ± 0.4; 3.3 ± 0.4; 3.4 ± 0.4; 2.8 ± 0.4), thickening (0 ± 0; 0.4 ± 0.6; 1.2 ± 0.4; 2 ± 0.4; 2.8 ± 0.4; 3.3 ± 0.4; 3.7 ± 0.4) and skin scaling (0 ± 0; 0 ± 0; 1.1 ± 0.3; 1.6 ± 0.4; 2.6 ± 0.4; 3.2 ± 0.5; 3.5 ± 0.3) scores; (**b**) PASI scores (0 ± 0; 1.5 ± 0.6; 4.4 ± 0.6; 6.2 ± 0.8; 8.7 ± 0.6; 8.7 ± 0.6; 9.9 ± 0.7). The results are presented as mean score ± SD; *n* = 28 (*n* = number of mice; D = day); (**c**) H&E staining of the back skin samples provided from normal mouse (control group); (**d**) H&E staining of the back skin samples provided from IMQ-induced mouse (Ps group). IMQ-based cream induces hyperkeratosis, parakeratosis, acanthosis and elongation of rete ridges.

The experimental model of psoriatic dermatitis was performed to evaluate the effect of the IgY treatment on Ps-specific skin and systemic lesions. The murine model of psoriatic dermatitis previously described [43,44,47], used IMQ-based cream applied on the back skin area for 6 consecutive days in order to induce an extensive psoriatic-like reaction. Animals were monitored daily, and the severity of skin inflammation induced by applying IMQ-based cream was assessed based on daily scores of erythema, thickening and skin scaling, PASI score, splenomegaly evaluation and histopathological assessment. Erythema, thickening and scaling of the back skin were daily scored on a 0–4 scale (0—no change, 1—mild change, 2—marked change; 3—significant change, 4—severe change) and the evolution of these scores is shown in Figure 1a. Table S1 presents the individual PASI scores for all the animals within the groups.

Starting with day 2, signs of inflammation were visible and increased in intensity until the end of the application. The skin on the back region of the mice began to show signs of erythema, thickening, and scaling that became visible from day 3 of the experiment. As a measure of the disease severity, a modified PASI score (0–12 scale) was calculated daily, by summing erythema, thickening and skin scaling daily scores. The PASI cumulative score had a progressive evolution, reaching high values at the end of the applications (Figure 1b).

Skin inflammation induced by IMQ-based cream was histopathologically assessed. Skin samples harvested from all groups were collected at the end of experiment, fixed in 10% buffered formaldehyde and incorporated into paraffin; the paraffin blocks were sectioned (5 µm thick sections), stained with hematoxylin-eosin and examined by pathologist. Histopathological evaluation revealed hyperkeratosis, parakeratosis, acanthosis and elongation of the red ridges, histopathological features typical for human psoriatic lesions (Figure 1d). None of these features were observed in healthy mice (control group) (Figure 1c). Individual data for the assessment of all histological parameters and individual mice are presented in Table S2 (Supplementary Material).

### 3.2. IgY Treatment-Induced Changes in Experimental Murine Model of Psoriatic Dermatitis

For naturally remitted Ps group, the mice were allowed to heal naturally and erythema, thickening and skin scaling were daily observed in order to appreciate the day when the effects produced by applying of IMQ-based cream disappeared. The mice were weighed at the beginning of the experiment (day 1), after IMQ-based cream treatment (day 7) and before sacrifice—day 22—when the natural remission of the IMQ-skin effects was noticed.

For IgY-treated Ps group, mice received a dose of IgY by gavage for 5 consecutive days, The evolution of the three in vivo parameters (erythema, thickening and skin scaling) was monitored and we established that the skin healing took place in day 20, 2 days earlier than naturally remitted Ps group. The body weight was recorded at the beginning of the experiment (day 1), day 7 (beginning of IgY treatment), at the end of IgY treatment (day 12) and before sacrifice—day 20—when it was macroscopically appreciated that the IMQ-skin effects disappeared.

**Figure 2:**
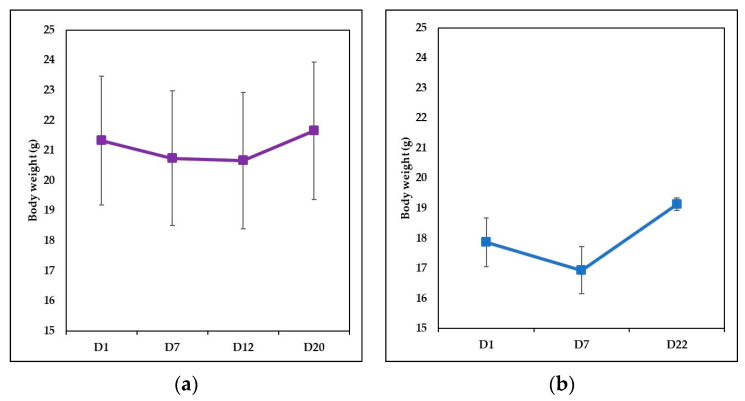
Evolution of the body weight (**a**) Evolution of the body weight for IgY-treated Ps group (*n* = 12)-day 1 (21.33 ± 2.14), day 7 (20.74 ± 2.24), day 12 (20.66 ± 2.27), day 20 (21.65 ± 2.28) (**b**) Evolution of the body weight for naturally remitted Ps group (*n* = 8)-day 1 (17.87 ± 0.81), day 7 (16.93 ± 0.78), day 22 (19.13 ± 0.21). The results are presented as mean ± SD; *n* = number of mice; D = day.

Body weight (Figure 2a,b) revealed a decrease after the IMQ topical application (day 7) for both IgY-treated Ps group and naturally remitted Ps group. For the IgY-treated Ps group (Figure 2a) the values decrease until the end of the IgY treatment and at the end of the experiment, for both experimental groups, weight increases were recorded.

**Figure 3:**
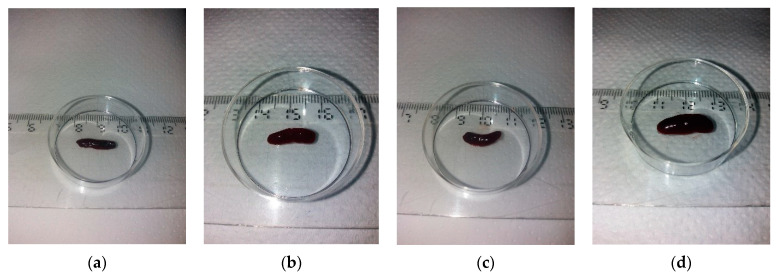

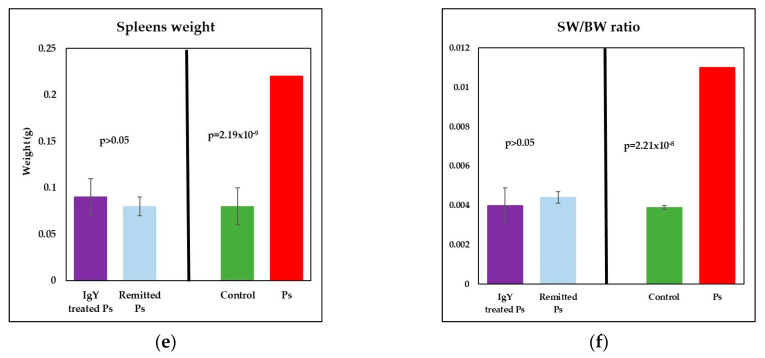
Representative images for the assessment of splenomegaly reduction. Spleens harvested from (**a**) mouse from IgY – treated Ps group; (**b**) mouse naturally remitted Ps group; (**c**) mouse from control group; (**d**) mouse from Ps group; (**e**) The weight of the spleens for IgY – treated Ps group (*n* = 12) (0.09 ± 0.02) as compared to naturally remitted Ps group (*n* = 8) (0.08 ± 0.01), control group (*n* = 8) (0,08 ± 0.01) and Ps group (*n* = 8) (0.22 ± 0.02); (**f**) SW/BW ratio for IgY-treated Ps group (*n* = 12) (0.0040 ± 0.0009) as compared to naturally remitted Ps group (*n* = 8) (0.0044 ± 0.0003), control group (*n* = 8) (0.0040 ± 0.0009) and Ps group (*n* = 8) (0.011 ± 0.001). The results are presented as mean ± SD; *n* = number of mice).

In order to assess the involvement of the triggered Ps on the secondary immune organs, splenomegaly evaluation was performed. Spleens were weighed separately and the ratio between spleen weight (SW) and body weight (BW) was calculated. Spleen weight was significantly higher in Ps group (0.22 ± 0.02, *p* = 2.19 × 10^−9^) as compared to controls (0.08 ± 0.01) (Figure 3e). The measurements also showed that in IMQ-mice, SW/BW ratio is almost 3 times higher than in healthy mice (0.011 ± 0.001 versus 0.0039 ± 0.0002, *p* = 2.21 × 10^−8^) (Figure 3f).

The SW/BW parameter was evaluated also for IgY-treated group. As mentioned above, a marked splenomegaly was noticed in IMQ-based experimental model of psoriatic dermatitis, namely spleen weights and SW/BW were about three times higher for Ps group as compared to control group. In the IgY-treated group there is a clear reduction of the splenomegaly. Figure 3a–d presents the macroscopic images of spleens and Figure 3e shows the mean values of spleen weights for all experimental groups.

As a measure of splenomegaly, SW/BW ratio was calculated for all groups and IgY-treated Ps group and naturally remitted Ps group were compared to control and Ps groups (Figure 3f).

At the end of the experiment, values for both spleen weight and SW/BW ratio after IgY treatment were identical or statistically equivalent to the values recorded for the control group Additionally, there are no statistically significant differences between IgY-treated Ps group and naturally remitted Ps group, for both spleen weight and SW/BW ratio. Thus, the splenomegaly observed after the induction of Ps (respectively, after applying the IMQ-based cream for 6 consecutive days) was completely remitted.

### 3.3. IgY Treatment-Induced Changes in Lymphocyte Distribution in Peripheral Blood and Spleen Cell Suspensions in Experimental Murine Model of Psoriatic Dermatitis

To evaluate the immune cells populations/subpopulations that follow the clinical improvement of the induced psoriasis lymphocyte immunophenotyping was performed by flow cytometry from both peripheral blood and spleen cell suspensions. For all experimental groups we quantified T lymphocytes (CD3ε^+^), with T-helper (CD4^+^CD8^−^) and T-suppressor/cytotoxic (CD8a^+^CD4^−^) subsets, B lymphocytes (CD3ε^−^CD19^+^) and NK cells (CD3ε^−^NK1.1^+^).

**Figure 4:**
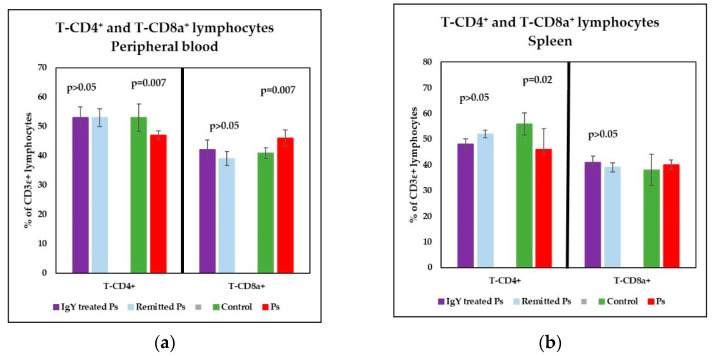
Distribution of T-CD4^+^ and T-CD8a^+^ lymphocytes in peripheral blood and spleen cell suspension. (**a**) Peripheral blood-distribution of T-CD4^+^ and T-CD8a^+^ lymphocytes for IgY-treated Ps group (*n* = 12) (53 ± 3.6 and 42 ± 3.3) as compared to naturally remitted Ps group (*n* = 8) (53 ± 3 and 39 ± 2.4), control group (*n* = 8) (53 ± 4.7 and 41 ± 1.8) and Ps group (*n* = 8) (47 ± 1.5 and 46 ± 2.8); (**b**) Spleen cell suspension-distribution of T-CD4^+^ and T-CD8a^+^ lymphocytes for IgY-treated Ps group (*n* = 12) (48 ± 2.2 and 41 ± 2.3) as compared to naturally remitted Ps group (*n* = 8) (52 ± 1.5 and 39 ± 1.8), control group (*n* = 8) (56 ± 4.3 and 38 ± 6.1) and Ps group (*n* = 8) (46 ± 8.1 and 40 ± 1.9). The results (% of CD3ε^+^ lymphocytes) are presented as mean ± SD; *n* = number of mice).

**Figure 5:**
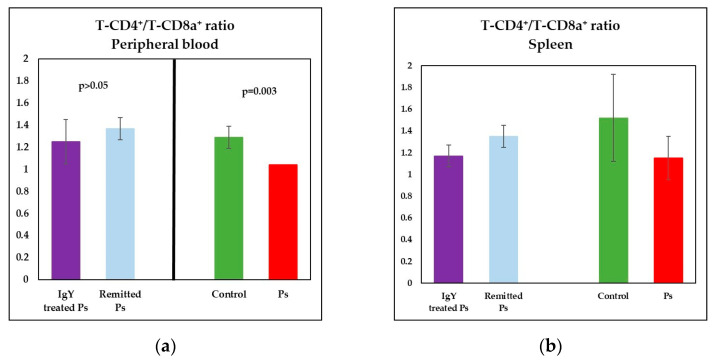
Distribution of T-CD4^+^/T-CD8a^+^ ratio in peripheral blood and spleen cell suspension. (**a**) Peripheral blood-distribution of T-CD4^+^/T-CD8a^+^ ratio for IgY-treated Ps group (*n* = 12) (1.25 ± 0.2) as compared to naturally remitted Ps group (*n* = 8) (1.37 ± 0.1), control group (*n* = 8) (1.29 ± 0.2) and Ps group (*n* = 8) (1.04 ± 0.1); (**b**) Spleen cell suspension–distribution of T-CD4^+^/T-CD8a^+^ ratio for IgY-treated Ps group (*n* = 12) (1.17 ± 0.1) as compared to naturally remitted Ps group (*n* = 8) (1.35 ± 0.1), control group (*n* = 8) (1.52 ± 0.4) and Ps group (*n* = 8) (1.15 ± 0.2). The results are presented as mean ± SD; *n* = number of mice).

**Figure 6:**
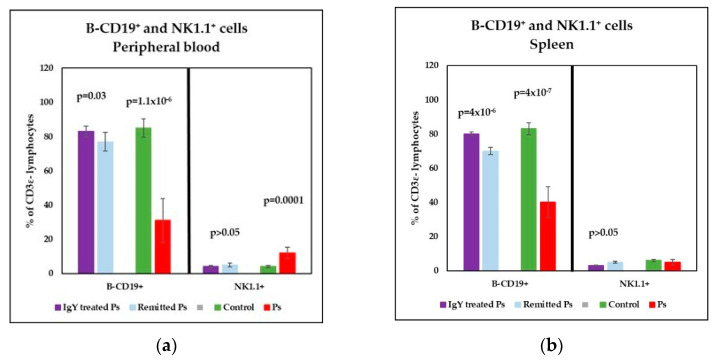
Distribution of B-CD19^+^ and NK1.1^+^ cells in peripheral blood and spleen cell suspension. (**a**) Peripheral blood-distribution of B-CD19^+^ and NK1.1^+^ cells for IgY-treated Ps group (*n* = 12) (83 ± 3.2 and 4 ± 0.7) as compared to naturally remitted Ps group (*n* = 8) (77 ± 5.5 and 5 ± 1.1), control group (*n* = 8) (85 ± 5.3 and 4 ± 0.7) and Ps group (*n* = 8) (31 ± 12.8 and 12 ± 3.3); (**b**) Spleen cell suspension–distribution of B-CD19^+^ and NK1.1^+^ cells for IgY-treated Ps group (80 ± 1 and 3 ± 0.4) as compared to naturally remitted Ps group (70 ± 2.1 and 5 ± 0.5), control group (83 ± 3.6 and 6 ± 0.8) and Ps group (40 ± 9 and 5 ± 1.8). The results (% of CD3ε^−^ lymphocytes) are presented as mean ± SD; *n* = number of mice).

Therefore, a statistically significant lower percentages of T-CD4+ (*p* = 0.007) and signifi-cantly increased of T-CD8a+ lymphocytes (*p* = 0.007 vs) were obtained (Figure 4a). As a consequence of the changes observed in T subsets distribution, the T-CD4+/T-CD8+ ratio was decreased in Ps group as compared to control group (*p* = 0.003) (Figure 5a). Also, a decreased percentage of B lymphocytes (*p* = 1.1 × 10^−6^) and a significantly increased of NK1.1+ cells percentages (*p* = 0.0001) were registered (Figure 6a).

The main changes observed in spleen cell suspensions were statistically significant in Ps group, namely lower percentages of T-CD4^+^ (*p* = 0.02) and B lymphocytes (*p* = 4 × 10^−7^), (Figure 4b and Figure 6b). T-CD4^+^/T-CD8^+^ ratio is decreased in Ps mice as compared to control group but not statistically significant (Figure 5b).

Analysis of T-CD4^+^ and T-CD8a^+^ lymphocyte subsets in peripheral blood revealed normalization of mean percentage values of these parameters for both IgY-treated Ps group and naturally remitted Ps group (Figure 4a). There were statistically significant differences between the values of T-CD4^+^ and T-CD8a^+^ subsets for Ps group and IgY-treated Ps group (*p* = 0.002 for T-CD4^+^ and *p* = 0.04 for T-CD8a^+^), and naturally remitted Ps group (*p* = 0.002 for T-CD4^+^ and *p* = 0.006 for T-CD8a^+^), respectively, comparable to the differences observed between Ps group and controls for the investigated parameters. No statistically significant differences were observed between IgY-treated Ps group and naturally remitted Ps group for these T-subsets. Furthermore, no statistically significant differences were observed between IgY-treated Ps group and control group for T-CD4^+^ and T-CD8a^+^ subsets (*p* > 0.05), thus statistically underlining the normalization of these values after IgY treatment.

Data obtained for T-CD4^+^ and T-CD8a^+^ subpopulations in spleen cell suspensions revealed a tendency to normalization for both IgY-treated Ps group and naturally remitted Ps group (Figure 4b). For T-CD8a^+^ lymphocytes, no statistically significant differences were observed between the IgY-treated Ps group and control group. Although the values of T-CD4^+^ subset obtained for IgY-treated Ps group were significantly lower than control (*p* = 0.006), no statistically significant differences were observed between the IgY-treated Ps group and naturally remitted Ps group for T-CD4^+^ and T-CD8a^+^ subsets.

Analysis of the T-CD4^+^/T-CD8a^+^ ratio in the peripheral blood revealed the normalization of the mean values for both IgY-treated Ps group and naturally remitted Ps group (Figure 5a). There were statistically significant differences between Ps group and naturally remitted Ps groups (*p* = 0.0003), respectively IgY-treated Ps group (*p* = 0.01), and no statistically significant differences between control group and IgY-treated Ps group, respectively naturally remitted Ps group. The value of the T-CD4^+^/T-CD8a^+^ ratio for IgY-treated group was almost identical to control group (1.25 vs. 1.29).

A tendency of normalization of T-CD4^+^/T-CD8a^+^ ratio values was also noticed in spleen cell suspensions for both experimental groups. Although the values obtained for IgY-treated Ps group were significantly lower than control (*p* = 0.04), no statistically significant differences were observed between the IgY-treated Ps group and naturally remitted Ps group for T-CD4^+^/T-CD8a^+^ ratio.

Analysis of B-CD19^+^ and NK1.1^+^ cells in peripheral blood revealed normalization of these parameters for both IgY-treated Ps group and naturally remitted Ps group (Figure 6a). Statistically significant differences were observed between the Ps group and the naturally remitted Ps group (*p* = 0.0003 for B-CD19^+^ and *p* = 0.01 for NK1.1^+^), respectively, and the IgY-treated Ps group (*p* = 1 × 10^−8^ for B-CD19^+^ and *p* = 9.8 × 10^−6^ for NK1.1^+^). Although for both experimental groups B-CD19^+^ and NK1.1^+^ normalization in peripheral blood was noticed, it is important that the values normalization obtained for IgY-treated Ps group is more pronounced, and the days necessary for skin healing is reduced compared to the naturally remitted Ps group. The normalization of values for the IgY-treated group is also supported by the fact that no statistically significant differences were obtained between the IgY-treated group and controls.

The main change observed in the cellular population of the spleen was the normalizing of B-CD19^+^ cells revealed by a significant increase of B lymphocyte percentages in both IgY-treated Ps group and naturally remitted Ps group (Figure 6b). Statistically significant differences were observed between Ps group and the naturally remitted Ps group (*p* = 0.0006), respectively IgY-treated Ps (*p* = 2.1 × 10^−9^) group, differences were also noticed between the Ps group and controls (*p* = 4 × 10^−7^). Although for both experimental groups, the normalization of B-CD19^+^ values from spleen cell suspension were observed, once more the normalization is enhanced for IgY-treated Ps group, and less healing days necessary compared to naturally remitted Ps group. No statistically significant differences were obtained for B-CD19^+^ lymphocytes between IgY-treated Ps group and controls (*p* > 0.05), the values obtained being comparable. For NK1.1^+^ cells, there is a tendency to normalize their values, and it was more pronounced in this case for the naturally remitted Ps group, but no statistically significant differences were observed between the IgY-treated Ps group and naturally remitted Ps group.

### 3.4. IgY Treatment-Induced Changes in NK Phenotype in Peripheral Blood and Spleen Cell Suspensions in Experimental Murine Model of Psoriatic Dermatitis

The expressions on NK cells of CD49b, CD11b, CD43, CD27, KLRG1-maturation markers, on CD69, CD28, CD11c, NKp46-activation markers, respectively, were quantified for all experimental groups.

**Figure 7:**
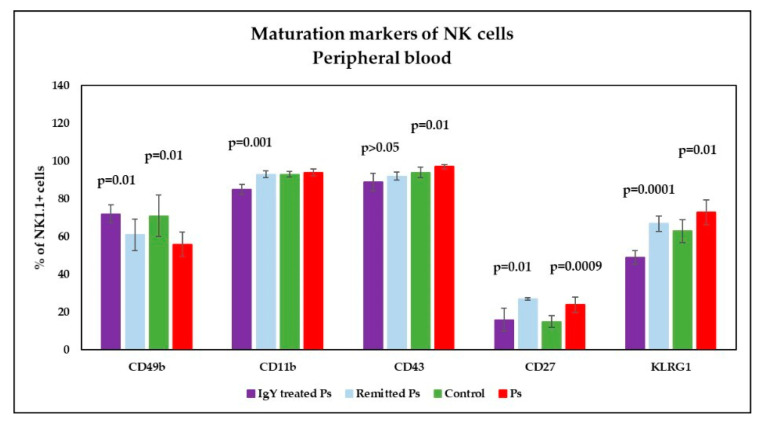
Expression of maturation markers on NK cells in peripheral blood. Expression of CD49b, CD11b, CD43, CD27 and KLRG1 levels on NK1.1^+^ cells for IgY-treated Ps group (*n* = 12) (72 ± 4.7; 85 ± 2.7; 89 ± 4.6; 16 ± 6.1; 49 ± 3.7) as compared to naturally remitted Ps group (*n* = 8) (61 ± 8.3; 93 ± 1.8; 92 ± 2.1; 27 ± 0.8; 67 ± 4.1), control group (*n* = 8) (71 ± 11; 93 ± 1.5; 94 ± 2.7; 15 ± 3; 63 ± 6) and Ps group (*n* = 8) (56 ± 6.5; 94 ± 1.8; 97 ± 1.3; 24 ± 4.1; 73 ± 6.6). The results (% of NK1.1^+^ cells) are presented as mean ± SD; *n* = number of mice).

**Figure 8:**
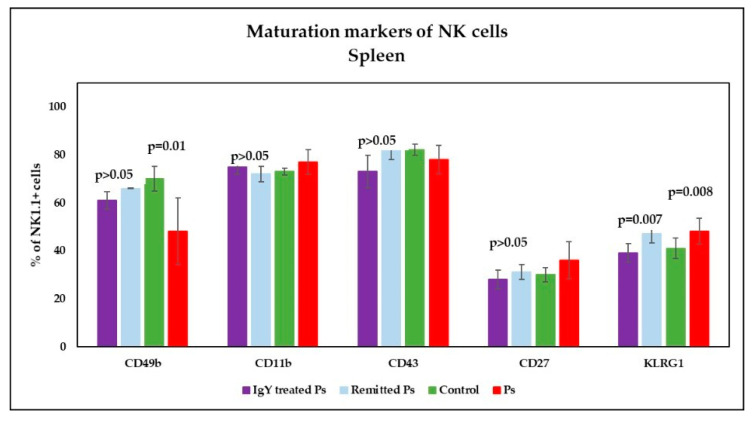
Expression of maturation markers on NK cells in spleen cell suspensions. Expression of CD49b, CD11b, CD43, CD27 and KLRG1 levels on NK1.1^+^ cells for IgY-treated Ps group (*n* = 12) (61 ± 3.7; 75 ± 3.1; 73 ± 6.8; 28 ± 3.9; 39 ± 3.9) as compared to naturally remitted Ps group (*n* = 8) (66 ± 0.2; 72 ± 3.2; 82 ± 4.1; 31 ± 3.1; 47 ± 3.7), control group (*n* = 8) (70 ± 5.1; 73 ± 1.4; 82 ± 2.3; 30 ± 2.9; 41 ± 4.3) and Ps group (*n* = 8) (48 ± 13.9; 77 ± 5.2; 78 ± 6; 36 ± 7.8; 48 ± 5.4). The results (% of NK1.1^+^ cells) are presented as mean ± SD; *n* = number of mice).

The analysis of maturation markers in peripheral blood (Figure 7) showed a significant tendency to increase their expression on NK cells as compared to control group and the differences between the experimental groups were statistically significant (*p* = 0.01; *p* = 0.0009; *p* = 0.01). The level of CD49b on NK cells is significant reduced in Ps group. The percentages of NK1.1^+^CD11b^+^ cells in Ps group are higher than controls, but without statistical significance. In spleen cell suspension (Figure 8), analysis of maturation markers revealed the same tendency of variation: increased values for CD11b, CD27, KLRG1 levels on NK cells and lower values for CD49b and CD43 in Ps mice as compared to controls. Only for CD49b and KLRG1 the differences were statistically significant (*p* = 0.01; *p* = 0.008).

**Figure 9:**
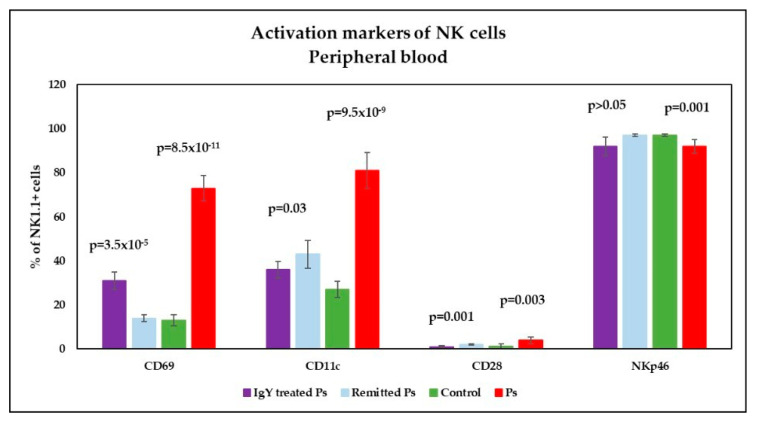
Expression of activation markers on NK cells in peripheral blood. Expression of CD69, CD11c, CD28 and NKp46 levels on NK1.1^+^ cells for IgY-treated Ps group (*n* = 12) (31 ± 4; 36 ± 3.8; 1 ± 0.5; 92 ± 4.2) as compared to naturally remitted Ps group (*n* = 8) (14 ± 1.6; 43 ± 6.4; 2 ± 0.3; 97 ± 0.6), control group (*n* = 8) (13 ± 2.6; 27 ± 3.7; 1.2 ± 1.2; 97 ± 0.5) and Ps group (*n* = 8) (73 ± 5.7; 81 ± 8.1; 4 ± 1.5; 92 ± 3.2). The results (% of NK1.1^+^ cells) are presented as mean ± SD; *n* = number of mice).

**Figure 10:**
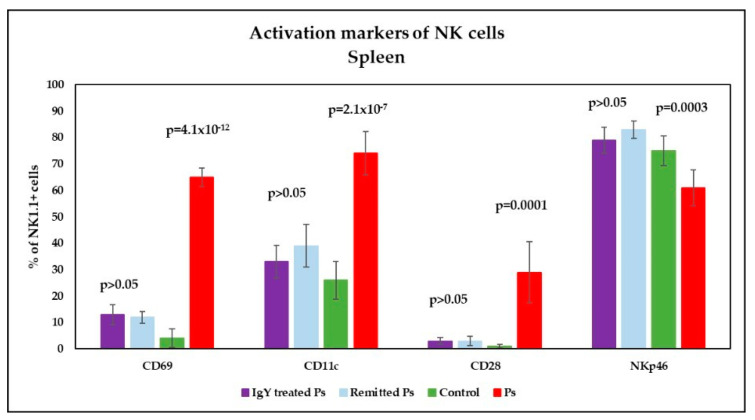
Expression of activation markers on NK cells in spleen cell suspensions. Expression of CD69, CD11c, CD28 and NKp46 levels on NK1.1^+^ cells for IgY-treated Ps group (*n* = 12) (13 ± 3.8; 33 ± 6.2; 3 ± 1.2; 79 ± 5) as compared to naturally remitted Ps group (*n* = 8) (12 ± 2.2; 39 ± 8.1; 3 ± 1.8; 83 ± 3.2), control group (*n* = 8) (4 ± 3.5; 26 ± 7.1; 1 ± 0.7; 75 ± 5.7) and Ps group (*n* = 8) (65 ± 3.5; 74 ± 8.2; 29 ± 11.5; 61 ± 6.8). The results (% of NK1.1^+^ cells) are presented as mean ± SD; *n* = number of mice).

Analysis of CD69, CD11c and CD28 (activation markers) on NK1.1^+^ cells in peripheral blood revealed significantly increased values in Ps group *p* = 8.5 × 10^−11^; *p* = 9.5 × 10^−9^; *p* = 0.003) compared to controls; the expression of NKp46 on NK1.1^+^ cells is lower in Ps mice as compared to controls and the differences between the experimental groups were statistically significant (*p* = 0.001) (Figure 9). We found the same tendency of variation for activation markers in spleen cell suspensions (Figure 10): significantly increased values for CD69, CD11c and CD28 in Ps group (*p* = 4.1 × 10^−12^; *p* = 2.1 × 10^−7^; *p* = 0.0001) compared to controls; the expression of NKp46 on NK1.1^+^ cells is lower in Ps mice as compared to controls, and the differences between the experimental groups were statistically significant (*p* = 0.0003).

Analysis of NK cell maturation markers in peripheral blood (Figure 7) revealed a normalization of values for NK1.1^+^CD49^+^ and NK1.1^+^CD27^+^ cells in the IgY-treated Ps group. As there are statistically significant differences (*p* = 0.01 and *p* = 0.0009) obtained for these parameters between the Ps and control groups, after application of the IgY treatment we did not find statistical differences when comparing the values obtained for IgY-treated Ps group and controls. The expression of CD11b, CD43 and KLRG1 markers on NK1.1^+^ cells is significantly lower for IgY-treated Ps group as compared to control group. For the naturally remitted Ps group, the values for CD49b, CD11b, CD43 and KLRG1 are normalized when comparing the values obtained for naturally remitted Ps group and controls. The expression of CD27 on NK1.1^+^ cells was still significantly increased (*p* = 0.0003) for the naturally remitted Ps group, being almost equal to that obtained for the Ps group.

In the spleen (Figure 8), the analysis of CD11b, CD27 and KLRG1 maturation markers revealed the normalization of their expression on NK cells following IgY treatment when comparing to control group. CD43 expression on NK cells decreased after IgY treatment. There were no statistically significant differences between IgY-treated Ps group and naturally remitted Ps group for CD49b, CD11b, CD43 and CD27 on NK cells.

Analysis of the expression of activation markers on NK1.1^+^ cells in peripheral blood after IgY treatment revealed the normalization of CD28 values when compared to controls. For CD69 and CD11c levels in IgY-treated Ps group we observed a significant decreasing trend compared to Ps group but the expressions of these NK markers are significantly increased compared to control group (Figure 9). Although there is no statistically significant difference between the IgY-treated Ps group and naturally remitted Ps group for NKP46 expression, its expression on NK cells after IgY treatment is comparable to Ps group, namely below normal limits. For naturally remitted Ps group, the values of all activation markers have normalized when comparing to controls, except for CD11c, whose expression is significantly increased compared to control group and IgY-treated Ps group (*p* = 0.001 and *p* = 0.03, respectively).

Analysis of the expression of CD69, CD11c and CD28 activation markers on NK1.1^+^ cells in spleen showed a pronounced decreasing trend for both IgY-treated mice and naturally remitted Ps group, toward normalization of their values (Figure 10). For CD11c expression, there is no statistically significant difference between IgY-treated Ps group and control group, while for naturally remitted Ps group, there are still significant differences (*p* = 0.03), when compared to controls. For all activation markers there are no statistically significant difference between IgY-treated Ps group and naturally remitted Ps group. NKp46 expression on NK cells have normalized in both IgY-treated group and naturally remitted Ps group as compared to the control group. Normalization of NKp46 values is more evident after IgY treatment.

## 4. Discussion

Psoriasis affecting the health of numerous individuals world-wide has a multifactorial pathogenesis and the exact triggering factor remains still unclear, As the skin is the major human organ with multiple functions, Ps instalment would trigger complex systemic disturbances. Alterations in the skin and intestinal microbiome are involved in the pathogenesis of psoriasis, therefore microbiome restoration becomes a promising preventive/therapy strategy in psoriasis [49].

Several years ago, it was shown that the overall microbial diversity is increased in the psoriatic plaque [50]. More recent studies proclaim an abnormal gut/skin microbiome as a potential driving force of systemic inflammation underlying Ps. It is hypothesized a gut-skin axis to be involved in Ps etiology as gut microbiota dysbiosis may alter systemic immunity and diminishes skin’s physiological functions [42,51]. Regarding therapy strategies, Ps treatment resembles bowel disease and could implicate appropriate antibiotics to restore a normal flora, and also the use of prebiotics might be an alternative avenue to explore [52]. Therefore, aiding current therapies with adjuvant compounds becomes a necessity. As IgY is gaining new therapeutical potential in the anti-viral and anti-bacterial fight and acknowledging all the accumulated data, we have initiated in a psoriasis experimental model an adjuvant therapy using oral IgY developed against several pathogenic bacteria to evaluate the potency to alleviate the psoriatic lesions and to restore the cellular immune-related mechanisms.

The IMQ-induced psoriasiform dermatitis model [47] represents one of the most used inducible systems in studying Ps due to its reduced cost, rapid induction of skin inflammation and high reproducibility. Topical application of IMQ in animal models induce the formation of cutaneous lesions similar with human Ps plaque, [53,54,55]. group [53,54], Splenomegaly, as an indicator of intense lymphocyte activation, was observed in all experimental groups in which psoriatic dermatitis was induced. As recently published, splenomegaly is a characteristic of this animal model [56] and it is an indicator that although the induction of lesions was topical there is a systemic immune response. Following oral therapy with IgY or naturally healing, the values of spleen weight and SW / BW ratio were identical to the control group values. Practically, the splenomegaly installed after 6 consecutive days of IMQ-based cream topical application was completely remitted, for both experimental groups.

As reported in Ps patients [57], peripheral and spleen immune cell deregulations were found. As previously reported also by other groups [58] lower percentages of T-CD4^+^ and B lymphocytes, while the percentages of T-CD8a^+^ lymphocytes and NK1.1^+^ cells were significantly increased. As a consequence, the T-CD4^+^/T-CD8^+^ ratio was significant decreased in Ps mice. The main changes observed in spleen cell suspensions were statistically significant, namely lower percentages of T-CD4^+^ and B lymphocytes for Ps group as compared to controls. T-CD4^+^/T-CD8^+^ ratio is decreased in Ps mice, but the differences between the experimental groups were not statistically significant. The values obtained for these immunological parameters are comparable to the results published by our research team for psoriatic dermatitis mice model in which the IMQ-based cream was applied for 5 consecutive days [44] and with other group’s results [59].

Analysis of T-CD4^+^ and T-CD8a^+^ lymphocyte subsets in peripheral blood revealed normalization of these parameters for IgY-treated Ps, naturally remitted Ps and control groups. T-CD8a^+^ lymphocytes, identified in spleen cell suspensions in the IgY-treated Ps group are identical to the control group. Although the values of T-CD4^+^ subset obtained for IgY-treated Ps group were significantly lower than control (*p* = 0.006), no statistically significant differences were observed between the IgY-treated Ps group and naturally remitted Ps group for T-CD4^+^ subset. Recent findings have shown that T-CD8^+^ cells are involved in psoriasiform skin inflammation and that memory T cells are involved in the pathogenesis of psoriasis, especially its recurrence. Therefore, normalization of these values brings clear clinical benefit [60].

As expected, the T-CD4^+^/T-CD8a^+^ ratio in peripheral blood also revealed the normalization pattern of IgY-treated Ps group compared to naturally remitted Ps or control group. A tendency of normalization was also noticed in spleen cell suspensions for both experimental groups. Although T-CD4^+^/T-CD8a^+^ ratio for IgY-treated Ps group were significantly lower than control (*p* = 0.04), no statistically significant differences were observed between the IgY-treated Ps group and naturally remitted Ps group.

Even though B-CD19^+^ and NK1.1^+^ normalization in peripheral blood was noticed the normalization for IgY-treated Ps group is more pronounced.

The main change observed in the spleen cell suspension was the normalizing of B–CD19^+^ cells by significant increase of B lymphocyte percentages in both IgY-treated Ps group and naturally remitted Ps group. B cells have an important role in the protection against different infectious and inflammatory diseases, but there are very few reports on B lymphocytes involvement in Ps. The regulatory sub-population of B cells, B_regs_ were found decreased in Ps patients [61] and moreover, it was shown that B_regs_ may positively influence the course of Ps by producing IL-10 [62,63]. Therefore, the B cells increase that we have noticed in the treated group could account for the clinical improvement of the induced Ps.

For NK1.1^+^ cells, there is a tendency to normalize the values, in the naturally remitted Ps group, with no statistically significant differences when compared to IgY-treated Ps group. The role of NK cells in Ps development is not fully elucidated. Although NK cells are recruited in human psoriatic lesions and in the induced Ps in mice, the studies regarding NK cells involvement in Ps do not abound [64]. The level of maturation marker CD49b on NK cells is significantly reduced in the Ps group. In spleen cell suspension, analysis of maturation markers revealed the same tendency of variation: increased values for CD11b, CD27, KLRG1 levels on NK cells and lower values for CD49b and CD43 in Ps mice as compared to controls. Only for CD49b and KLRG1 the differences were statistically significant.

Analysis of activation markers CD69, CD11c and CD28 on NK1.1^+^ cells in peripheral blood revealed significantly increased values in Ps group compared to controls; the expression of NKp46 on NK1.1^+^ cells is lower in Ps mice as compared to controls, and the differences between the experimental groups were statistically significant. Published studies report controversial results regarding NK cells in Ps and the matter is still subject of debate [65,66,67].

We found the same tendency of variation for activation markers in spleen cell suspensions, namely significantly increased values for CD69, CD11c and CD28 in Ps group compared to controls along with decreased expression of NKp46 on NK1.1+, with statistically significant differences. As a major activating receptor, NKp46, is an NK cell specific surface marker involved in all NK physiological immune processes [68] therefore an indicator that NK cells are mis-functioning due to the induced Ps.

In IgY-treated Ps group, NK cell maturation markers assessed in the peripheral blood revealed a normalization of values for NK1.1^+^CD49^+^ and NK1.1^+^CD27^+^ cells. Thus, the statistically significant differences obtained for these parameters between the Ps and control groups subside after IgY treatment. For naturally remitted Ps group, the values for CD49b, CD11b, CD43 and KLRG1 are as well normalized when compared to controls. As already mentioned, it should be noted that the healing period was longer for naturally remitted Ps group compared to the IgY-treated group. The expression of CD27 on NK1.1^+^ cells was still significantly increased for the naturally remitted Ps group, being almost equal to that obtained for Ps group. NK maturation in periphery is characterized by an upregulation of CD11b, CD43, KLRG1, and Ly49 receptors, and a downregulation of CD27 [69,70], therefore we can speculate that even in clinically remitted psoriatic lesions the NK population remains alert to any psoriatic-dependent antigen.

In spleen cell suspension, analysis of CD11b, CD27 and KLRG1 maturation markers revealed the normalization of their expression on NK cells following IgY treatment compared to the control group. CD43 expression on NK cells decreased after IgY treatment. In contrast to the periphery, in the spleen, no statistically significant differences between IgY-treated Ps group and naturally remitted Ps group for CD49b, CD11b, CD43 and CD27 on NK cells was found. Yet again we can speculate that while NK residing in the secondary immune organs, such as the spleen, have normalized their parameters while in the periphery there are still populations that patrol in search of a psoriatic-like antigen.

Analysis of the expression of activation markers on NK1.1^+^ cells in peripheral blood after IgY treatment revealed the normalization of CD28 values when compared to controls. For CD69 and CD11c levels in IgY-treated Ps group we observed a significant decreasing trend compared to Ps group, but the expressions of these markers on NK cells are significantly increased compared to control group. Although there is no statistically significant difference between IgY-treated Ps group and naturally remitted Ps group for NKP46, its expression on NK cells after IgY treatment is comparable to the Ps group, namely below normal limits. For naturally remitted Ps group, the values of all activation markers have normalized, except for CD11c, whose expression is significantly increased compared to control group and IgY-treated Ps group. Several years ago, it was reported that the CD56^+^CD16^+^CD11c^+^ NK population are endowed with important characteristics such as IFN-γ production, tumor cell cytotoxicity and promotion of *γδ* T lymphocyte proliferation [71]. Therefore, in our experimental model NK cells retain their activation capacity as proven by CD11c expression.

In spleen cell suspensions analysis of the expression of CD69, CD11c and CD28 activation markers on NK1.1^+^ cells showed a pronounced decreasing trend for IgY-treated mice normalizing their values. For CD11c expression, there is no statistically significant difference between IgY-treated Ps group and control group (*p* > 0.05), while for naturally remitted Ps group, there are still significant differences. For all activation markers there are no statistically significant difference between IgY-treated Ps group and naturally remitted Ps group. NKp46 expression on NK cells have normalized in both IgY-treated group and naturally remitted Ps group as compared to the control group. Normalization of NKp46 values is more obvious after IgY treatment.

Study limitations. We acknowledge some limitations of our study. Thus, as human Ps is a complex auto-immune disease comprising, as presented, various triggering factors, the mice model is an induced one, therefore it misses probably more complex relations gut-skin interrelation. Another limitation of the study is that the IgY compound is designed for human ingestion as it is comprised out of IgY developed against antibiotic-resistant bacteria, therefore there could be bacterial strains that were missed in our mice model. Yet, in our model the Ps lesions subside earlier than the naturally remitted group probably as the utilized IgY is most probably restoring the digestive track microbiota. This limitation is overridden by the findings that mouse and human gut microbiota have similarity at the genus level [72]. Moreover, the mouse gut microbiota has similar functionality with the human one [73,74], therefore we can speculate in our experimental model that at least for some of the gut bacteria the IgY compound restored the microbiota inducing hence the solving of the induced psoriasis.

Perspectives. Far from being exhaustive, our work can open new perspective in Ps therapy. Therefore, we can foresee application in human psoriasis by first establishing the patients gut microbiota, then inoculating hens with the bacteria and isolating the raised IgY. Then, in conjunction with the standard psoriasis therapy, purified IgY can be ingested in doses matching the ones tested within our work. As shown, IgY preparations are non-allergenic and have high biocompatibility. Hence, one can imagine in the future adjuvant setting in which personalized IgY could aid the established therapy, alleviate the psoriatic lesions and improve the overall health status of the patient.

## 5. Conclusions

Ps affecting the health of numerous individuals worldwide has a multifactorial pathogenesis and recently it was shown that alterations in the skin and intestinal microbiome are involved in the pathogenesis of Ps, therefore microbiome restoration becomes a promising preventive/therapy strategy in this pathology. In our pre-clinical design study, using a mice model of induced psoriatic dermatitis, we have tested the proof-of-concept that IgY raised against pathological human bacteria resistant to antibiotics can alleviate psoriatic lesions and restore immune parameters. We pin-pointed that the IgY specific compound can be a possible pre-biotic alternative adjuvant in Ps. As IgY preparation can be raised against individualized microbiome using this compound can open also the personalized medicine domain in Ps.

## Data Availability

The datasets used and/or analyzed during current study are available from corresponding author on reasonable request.

## Supplementary Materials

The following are available online at https://www.mdpi.com/article/10.3390/jpm11090841/s1, Table S1: Individual experimental data for erythema (E), skin scaling (S), thickening (T) and PASI score. Table S2: Individual histological parameters for each mouse from groups control, naturally remitted, psoriasis and IgY treated (- represents the absence of the investigated parameter, + low presence; ++ medium presence; +++ intense presence; ++++ extremely high presence).

## Abbreviations

APC, Allophycocyanin; BD, Becton Dickinson; B_regs_, regulatory B cells; CD, Cluster of Differentiation; DC, Dendritic Cell; FACS, Fluorescence-Activated Cell Sorting; FBS, Fetal Bovine Serum; H&E, Haematoxylin-Eosin; FITC, Fluorescein Isothiocyanate; IFN, Interferon; Ig, Immunoglobulin; IL, Interleukin; IMQ, Imiquimod; JAK, Janus Kinase; K2-EDTA, Kalium 2 EthyleneDiamine TetraAcetate; KLRG1, Killer Cell Lectin-Like Receptor G1; NK, Natural Killer cells; PASI, Psoriasis Area Severity Index; PE, Phycoerythrin; PE/Cy, Phycoerythrin Complex with Cyanine; PerCP/Cy, Peridinin Chlorophyll Protein Complex with Cyanine; R, Receptor; RORγt, Retinoic acid receptor-related orphan nuclear receptor gamma t; RPMI, Roswell Park Memorial Institute; SD, Standard Deviation; SW/BW, Spleen Weight/Body Weight; Th, Helper T Cells; TLR, Toll-Like Receptor; TNF, Tumor Necrosis Factor; UV, Ultraviolet.

## References

[1] Guo R., Zhang T., Meng X., Lin Z., Lin J., Gong Y., Liu X., Yu Y., Zhao G., Ding X. (2019). Lymphocyte Mass Cytometry Identifies a CD3^−^CD4^+^ Cell Subset with a Potential Role in Psoriasis. JCI Insight.

[2] Nestle F.O., Kaplan D.H., Barker J. (2009). Psoriasis. N. Engl. J. Med..

[3] Parisi R., Symmons D.P.M., Griffiths C.E.M., Ashcroft D.M., Identification and Management of Psoriasis and Associated ComorbidiTy (IMPACT) Project Team (2013). Global Epidemiology of Psoriasis: A Systematic Review of Incidence and Prevalence. J. Investig. Dermatol..

[4] Parisi R., Iskandar I.Y.K., Kontopantelis E., Augustin M., Griffiths C.E.M., Ashcroft D.M. (2020). National, Regional, and Worldwide Epidemiology of Psoriasis: Systematic Analysis and Modelling Study. BMJ.

[5] Wolf P., Weger W., Patra V., Gruber-Wackernagel A., Byrne S.N. (2016). Desired Response to Phototherapy vs Photoaggravation in Psoriasis: What Makes the Difference?. Exp. Dermatol..

[6] Fry L., Baker B.S. (2007). Triggering Psoriasis: The Role of Infections and Medications. Clin. Dermatol..

[7] Naldi L. (2016). Psoriasis and Smoking: Links and Risks. Psoriasis.

[8] Wheatley R., Brooks J., Stumpf B., Boh E. (2017). Obesity, Diet, and Inflammation in Psoriasis. J. Psoriasis Psoriatic Arthritis.

[9] Farkas A., Kemény L. (2013). Alcohol, Liver, Systemic Inflammation and Skin: A Focus on Patients with Psoriasis. Skin Pharmacol. Physiol..

[10] Wolk K., Mallbris L., Larsson P., Rosenblad A., Vingård E., Ståhle M. (2009). Excessive Body Weight and Smoking Associates with a High Risk of Onset of Plaque Psoriasis. Acta Derm.-Venereol..

[11] Snast I., Reiter O., Atzmony L., Leshem Y.A., Hodak E., Mimouni D., Pavlovsky L. (2018). Psychological Stress and Psoriasis: A Systematic Review and Meta-Analysis. Br. J. Dermatol..

[12] Finlay A.Y., Khan G.K. (1994). Dermatology Life Quality Index (DLQI)—A Simple Practical Measure for Routine Clinical Use. Clin. Exp. Dermatol..

[13] Langley R.G., Ellis C.N. (2004). Evaluating Psoriasis with Psoriasis Area and Severity Index, Psoriasis Global Assessment, and Lattice System Physician’s Global Assessment. J. Am. Acad. Dermatol..

[14] Georgescu S.-R., Tampa M., Caruntu C., Sarbu M.-I., Mitran C.-I., Mitran M.-I., Matei C., Constantin C., Neagu M. (2019). Advances in Understanding the Immunological Pathways in Psoriasis. Int. J. Mol. Sci..

[15] Surcel M., Huica R., Constantin C., Ursaciuc C., Neagu M. (2017). Biomarkers Insights in Psoriasis-Regulatory Cytokines. Curr. Biomark..

[16] Gall J.S., Kalb R.E. (2008). Infliximab for the Treatment of Plaque Psoriasis. Biologics.

[17] Alwawi E.A., Mehlis S.L., Gordon K.B. (2008). Treating Psoriasis with Adalimumab. Ther. Clin. Risk Manag..

[18] Blauvelt A., Reich K., Lebwohl M., Burge D., Arendt C., Peterson L., Drew J., Rolleri R., Gottlieb A.B. (2019). Certolizumab Pegol for the Treatment of Patients with Moderate-to-Severe Chronic Plaque Psoriasis: Pooled Analysis of Week 16 Data from Three Randomized Controlled Trials. J. Eur. Acad. Dermatol. Venereol..

[19] Farhi D. (2010). Ustekinumab for the Treatment of Psoriasis: Review of Three Multicenter Clinical Trials. Drugs Today.

[20] Nogueira M., Torres T. (2019). Guselkumab for the Treatment of Psoriasis—Evidence to Date. Drugs Context.

[21] Witjes H., Khatri A., Diderichsen P.M., Mandema J., Othman A.A. (2020). Meta-Analyses of Clinical Efficacy of Risankizumab and Adalimumab in Chronic Plaque Psoriasis: Supporting Evidence of Risankizumab Superiority. Clin. Pharmacol. Ther..

[22] Blauvelt A., Sofen H., Papp K., Gooderham M., Tyring S., Zhao Y., Lowry S., Mendelsohn A., Parno J., Reich K. (2019). Tildrakizumab Efficacy and Impact on Quality of Life up to 52 Weeks in Patients with Moderate-to-Severe Psoriasis: A Pooled Analysis of Two Randomized Controlled Trials. J. Eur. Acad. Dermatol. Venereol..

[23] López-Ferrer A., Vilarrasa E., Puig L. (2015). Secukinumab (AIN457) for the Treatment of Psoriasis. Expert Rev. Clin. Immunol..

[24] Papp K.A., Leonardi C.L., Blauvelt A., Reich K., Korman N.J., Ohtsuki M., Paul C., Ball S., Cameron G.S., Erickson J. (2018). Ixekizumab Treatment for Psoriasis: Integrated Efficacy Analysis of Three Double-Blinded, Controlled Studies. Br. J. Dermatol..

[25] Foulkes A.C., Warren R.B. (2019). Brodalumab in Psoriasis: Evidence to Date and Clinical Potential. Drugs Context.

[26] Pandya V.B., Kumar S., Sachchidanand, Sharma R., Desai R.C. (2018). Combating Autoimmune Diseases With Retinoic Acid Receptor-Related Orphan Receptor-γ (RORγ or RORc) Inhibitors: Hits and Misses. J. Med. Chem..

[27] Bachelez H., Choon S.-E., Marrakchi S., Burden A.D., Tsai T.-F., Morita A., Turki H., Hall D.B., Shear M., Baum P. (2019). Inhibition of the Interleukin-36 Pathway for the Treatment of Generalized Pustular Psoriasis. N. Engl. J. Med..

[28] Schwartz D.M., Kanno Y., Villarino A., Ward M., Gadina M., O’Shea J.J. (2017). JAK Inhibition as a Therapeutic Strategy for Immune and Inflammatory Diseases. Nat. Rev. Drug Discov..

[29] Page K.M., Suarez-Farinas M., Suprun M., Zhang W., Garcet S., Fuentes-Duculan J., Li X., Scaramozza M., Kieras E., Banfield C. (2020). Molecular and Cellular Responses to the TYK2/JAK1 Inhibitor PF-06700841 Reveal Reduction of Skin Inflammation in Plaque Psoriasis. J. Investig. Dermatol..

[30] Zanin-Zhorov A., Weiss J.M., Trzeciak A., Chen W., Zhang J., Nyuydzefe M.S., Arencibia C., Polimera S., Schueller O., Fuentes-Duculan J. (2017). Cutting Edge: Selective Oral ROCK2 Inhibitor Reduces Clinical Scores in Patients with Psoriasis Vulgaris and Normalizes Skin Pathology via Concurrent Regulation of IL-17 and IL-10. J. Immunol..

[31] Vaclavkova A., Chimenti S., Arenberger P., Holló P., Sator P.-G., Burcklen M., Stefani M., D’Ambrosio D. (2014). Oral Ponesimod in Patients with Chronic Plaque Psoriasis: A Randomised, Double-Blind, Placebo-Controlled Phase 2 Trial. Lancet.

[32] Robbins K., Bissonnette R., Maeda-Chubachi T., Ye L., Peppers J., Gallagher K., Kraus J.E. (2019). Phase 2, Randomized Dose-Finding Study of Tapinarof (GSK2894512 Cream) for the Treatment of Plaque Psoriasis. J. Am. Acad. Dermatol..

[33] Rahman S., Van Nguyen S., Icatlo F.C., Umeda K., Kodama Y. (2013). Oral Passive IgY-Based Immunotherapeutics. Hum. Vaccines Immunother..

[34] Torché A.-M., Le Dimna M., Le Corre P., Mesplède A., Le Gal S., Cariolet R., Le Potier M.-F. (2006). Immune Responses after Local Administration of IgY Loaded-PLGA Microspheres in Gut-Associated Lymphoid Tissue in Pigs. Vet. Immunol. Immunopathol..

[35] Kovacs-Nolan J., Mine Y. (2012). Egg Yolk Antibodies for Passive Immunity. Annu. Rev. Food Sci. Technol..

[36] Akita E.M., Jang C.B., Kitts D.D., Nakai S. (1999). Evaluation of Allergenicity of Egg Yolk Immunoglobulin Y and Other Egg Proteins by Passive Cutaneous Anaphylaxis. Food Agric. Immunol..

[37] Vega C.G., Bok M., Vlasova A.N., Chattha K.S., Fernández F.M., Wigdorovitz A., Parreño V.G., Saif L.J. (2012). IgY Antibodies Protect against Human Rotavirus Induced Diarrhea in the Neonatal Gnotobiotic Piglet Disease Model. PLoS ONE.

[38] Xu Y., Li X., Jin L., Zhen Y., Lu Y., Li S., You J., Wang L. (2011). Application of Chicken Egg Yolk Immunoglobulins in the Control of Terrestrial and Aquatic Animal Diseases: A Review. Biotechnol. Adv..

[39] Constantin C., Neagu M., Diana Supeanu T., Chiurciu V., Spandidos D.A. (2020). IgY—Turning the Page toward Passive Immunization in COVID-19 Infection (Review). Exp. Ther. Med..

[40] Michael A., Meenatchisundaram S., Parameswari G., Subbraj T., Selvakumaran R., Ramalingam S. (2010). Chicken Egg Yolk Antibodies (IgY) as an Alternative to Mammalian Antibodies. Indian J. Sci. Technol..

[41] Yu M., Zhang R., Ni P., Chen S., Duan G. (2019). Helicobacter Pylori Infection and Psoriasis: A Systematic Review and Meta-Analysis. Medicina.

[42] de Jesús-Gil C., Sans-de San Nicolàs L., Ruiz-Romeu E., Ferran M., Soria-Martínez L., García-Jiménez I., Chiriac A., Casanova-Seuma J.M., Fernández-Armenteros J.M., Owens S. (2021). Interplay between Humoral and CLA+T Cell Response against Candida albicans in Psoriasis. Int. J. Mol. Sci..

[43] Visser M.J.E., Kell D.B., Pretorius E. (2019). Bacterial Dysbiosis and Translocation in Psoriasis Vulgaris. Front. Cell Infect. Microbiol..

[44] Surcel M., Huică R.-I., Munteanu A.N., Isvoranu G., Pîrvu I.R., Ciotaru D., Constantin C., Bratu O., Căruntu C., Neagu M. (2019). Phenotypic Changes of Lymphocyte Populations in Psoriasiform Dermatitis Animal Model. Exp. Ther. Med..

[45] Surcel M., Munteanu A.N., Huică R.-I., Isvoranu G., Pîrvu I.R., Constantin C., Bratu O., Căruntu C., Zaharescu I., Sima L. (2019). Reinforcing Involvement of NK Cells in Psoriasiform Dermatitis Animal Model. Exp. Ther. Med..

[46] Pătraşcu I.V., Chiurciu V., Chiurciu C., Topilescu G. (2014). Procedure to Obtain and Use Hen Egg Immunoglobulins (IgY).

[47] Pătraşcu I.V., Chiurciu V., Chiurciu C., Topilescu G. (2014). Method for Immunobiological Assay of Chicken Immunoglobulins Specific Activity.

[48] van der Fits L., Mourits S., Voerman J.S.A., Kant M., Boon L., Laman J.D., Cornelissen F., Mus A.-M., Florencia E., Prens E.P. (2009). Imiquimod-Induced Psoriasis-like Skin Inflammation in Mice Is Mediated via the IL-23/IL-17 Axis. J. Immunol..

[49] Chiurciu C., Chiurciu V., Sima L., Mihai I., Patrascu I.V. (2016). Production and Use of Personalized (Ovopatch) Hyperimmune Egg in the Treatment of Psoriasis.

[50] Chen L., Li J., Zhu W., Kuang Y., Liu T., Zhang W., Chen X., Peng C. (2020). Skin and Gut Microbiome in Psoriasis: Gaining Insight Into the Pathophysiology of It and Finding Novel Therapeutic Strategies. Front. Microbiol..

[51] Takemoto A., Cho O., Morohoshi Y., Sugita T., Muto M. (2015). Molecular Characterization of the Skin Fungal Microbiome in Patients with Psoriasis. J. Dermatol..

[52] Salem I., Ramser A., Isham N., Ghannoum M.A. (2018). The Gut Microbiome as a Major Regulator of the Gut-Skin Axis. Front. Microbiol..

[53] Haines Ely P. (2018). Is psoriasis a bowel disease? Successful treatment with bile acids and bioflavonoids suggests it is. Clin. Dermatol..

[54] Bocheńska K., Smolińska E., Moskot M., Jakóbkiewicz-Banecka J., Gabig-Cimińska M. (2017). Models in the Research Process of Psoriasis. Int. J. Mol. Sci..

[55] Hawkes J.E., Adalsteinsson J.A., Gudjonsson J.E., Ward N.L. (2018). Research Techniques Made Simple: Murine Models of Human Psoriasis. J. Investig. Dermatol..

[56] Yuan J., Ni G., Wang T., Mounsey K., Cavezza S., Pan X., Liu X. (2018). Genital Warts Treatment: Beyond Imiquimod. Hum. Vaccines Immunother..

[57] Jabeen M., Boisgard A.-S., Danoy A., El Kholti N., Salvi J.-P., Boulieu R., Fromy B., Verrier B., Lamrayah M. (2020). Advanced Characterization of Imiquimod-Induced Psoriasis-Like Mouse Model. Pharmaceutics.

[58] Alecu M., Ursaciuc C., Surcel M., Coman G., Ciotaru D., Dobre M. (2009). CD28 T-cell costimulatory molecule expression in pemphigus vulgaris. J. Eur. Acad. Dermatol. Venereol..

[59] Prietl B., Treiber G., Mader J.K., Hoeller E., Wolf M., Pilz S., Graninger W.B., Obermayer-Pietsch B.M., Pieber T.R. (2014). High-Dose Cholecalciferol Supplementation Significantly Increases Peripheral CD4^+^ Tregs in Healthy Adults without Negatively Affecting the Frequency of Other Immune Cells. Eur. J. Nutr..

[60] Shin S.-H., Kim H.-Y., Yoon H.-S., Park W.-J., Adams D.R., Pyne N.J., Pyne S., Park J.-W. (2020). A Novel Selective Sphingosine Kinase 2 Inhibitor, HWG-35D, Ameliorates the Severity of Imiquimod-Induced Psoriasis Model by Blocking Th17 Differentiation of Naïve CD4 T Lymphocytes. Int. J. Mol. Sci..

[61] Chen Y., Yan Y., Liu H., Qiu F., Liang C.-L., Zhang Q., Huang R.-Y., Han L., Lu C., Dai Z. (2020). Dihydroartemisinin Ameliorates Psoriatic Skin Inflammation and Its Relapse by Diminishing CD8+ T-Cell Memory in Wild-Type and Humanized Mice. Theranostics.

[62] Kahlert K., Grän F., Muhammad K., Benoit S., Serfling E., Goebeler M., Kerstan A. (2019). Aberrant B-Cell Subsets and Immunoglobulin Levels in Patients with Moderate-to-Severe Psoriasis. Acta Derm.-Venereol..

[63] Alrefai H., Muhammad K., Rudolf R., Pham D.A.T., Klein-Hessling S., Patra A.K., Avots A., Bukur V., Sahin U., Tenzer S. (2016). NFATc1 Supports Imiquimod-Induced Skin Inflammation by Suppressing IL-10 Synthesis in B Cells. Nat. Commun..

[64] Grän F., Kerstan A., Serfling E., Goebeler M., Muhammad K. (2020). Current Developments in the Immunology of Psoriasis. Yale J. Biol. Med..

[65] Polese B., Zhang H., Thurairajah B., King I.L. (2020). Innate Lymphocytes in Psoriasis. Front. Immunol..

[66] Kucuksezer U.C., Aktas Cetin E., Esen F., Tahrali I., Akdeniz N., Gelmez M.Y., Deniz G. (2021). The Role of Natural Killer Cells in Autoimmune Diseases. Front. Immunol..

[67] Luci C., Gaudy-Marqueste C., Rouzaire P., Audonnet S., Cognet C., Hennino A., Nicolas J.-F., Grob J.-J., Tomasello E. (2012). Peripheral Natural Killer Cells Exhibit Qualitative and Quantitative Changes in Patients with Psoriasis and Atopic Dermatitis. Br. J. Dermatol..

[68] Dunphy S.E., Sweeney C.M., Kelly G., Tobin A.M., Kirby B., Gardiner C.M. (2017). Natural Killer Cells from Psoriasis Vulgaris Patients Have Reduced Levels of Cytotoxicity Associated Degranulation and Cytokine Production. Clin. Immunol..

[69] Hadad U., Thauland T., Butte M., Porgador A., Martinez O., Krams S. NKp46 Clusters at the NK Cell Immune Synapse Regulate Specific Effector Functions. Proceedings of the 2015 American Transplant Congress.

[70] Abel A.M., Yang C., Thakar M.S., Malarkannan S. (2018). Natural Killer Cells: Development, Maturation, and Clinical Utilization. Front. Immunol..

[71] Ramírez-Ramírez D., Vadillo E., Arriaga-Pizano L.A., Mayani H., Estrada-Parra S., Velasco-Velázquez M.A., Pérez-Tapia S.M., Pelayo R. (2016). Early Differentiation of Human CD11c+NK Cells with Γδ T Cell Activation Properties Is Promoted by Dialyzable Leukocyte Extracts. J. Immunol. Res..

[72] Hugenholtz F., de Vos W.M. (2018). Mouse models for human intestinal microbiota research: A critical evaluation. Cell. Mol. Life Sci..

[73] Xiao L., Feng Q., Liang S., Sonne S.B., Xia Z., Qiu X., Li X., Long H., Zhang J., Zhang D. (2015). A catalog of the mouse gut metagenome. Nat. Biotechnol..

[74] Wang J., Lang T., Shen J., Dai J., Tian L., Wang X. (2019). Core Gut Bacteria Analysis of Healthy Mice. Front. Microbiol..

