# Supplementary material for: Unconventional Therapy with IgY in a Psoriatic Mouse Model Targeting Gut Microbiome
Source: J Pers Med. 2021 Aug 26;11(9):841. doi: 10.3390/jpm11090841 (PMC8466815; doi:10.3390/jpm11090841)
Supplement: Supplementary file 1 [file jpm-11-00841-s001.zip › jpm-1348971-supplementary.pdf]

Table S1. Individual experimental data for erythema (E), skin scaling (S), thickening (T) and PASI score.

	Time	Day 1				Day 2				Day 3				Day 4				Day 5				Day 6					Day 7		
Group	Mouse nb.	E	S	T	PASI	E	S	T	PASI	E	S	T	PASI	E	S	T	PASI	E	S	T	PASI	E	S	T	PASI	E	S	T	PASI
Naturally remitted Ps group	F1	0	0	0	0	1	0	0	1	2	1	1	4	2.5	1.5	2	6	3	2.5	3	8.5	3.5	3.5	3.5	8.5	3	4	4	11
	F2	0	0	0	0	1.5	0	0.5	2	2	1	1	4	3	1.5	2	6.5	3.5	2.5	3	9	3.5	3	3.5	9	3	3.5	4	10.5
	F3	0	0	0	0	1.5	0	1.5	3	2	1	1.5	4.5	2.5	1.5	2	6	4	2.5	2.5	9	4	3	3	9	3.5	3	3.5	10
	F4	0	0	0	0	1.5	0	0.5	2	2	1.5	1	4.5	3	1.5	2	6.5	3.5	2	3	8.5	3.5	3	3.5	8.5	3	3.5	4	10.5
	M1	0	0	0	0	1	0	0	1	2	1.5	0.5	4	3	1	1.5	5.5	3.5	2	2	7.5	4	3	3	7.5	3	3.5	3.5	10
	M2	0	0	0	0	1	0	0	1	1.5	1	1	3.5	2	1	2	5	3	3	3	9	3.5	3.5	3.5	9	2	3.5	4	9.5
	M3	0	0	0	0	1	0	0	1	2	1.5	1	4.5	2.5	1.5	2	6	3	2.5	3	8.5	3.5	3	4	8.5	2	3.5	4	9.5
	M4	0	0	0	0	1	0	0	1	2	1	1	4	3	1.5	2	6.5	3.5	3	3	9.5	4	3.5	3.5	9.5	2	4	4	10
Ps group	F1	0	0	0	0	1	0	0	1	2.5	1	1	4.5	2.5	1.5	1.5	5.5	3	2.5	3	8.5	3	3.5	3.5	8.5	2.5	4	3.5	10
	F2	0	0	0	0	1	0	0	1	2	1	0.5	3.5	2	2	1.5	5.5	3	2.5	2	7.5	3	3	3	7.5	2.5	3	3.5	9
	F3	0	0	0	0	1	0	0.5	1.5	1.5	1	1	3.5	2	2	2	6	3	3	3	9	3	4	3.5	9	2.5	3.5	4	10
	F4	0	0	0	0	1	0	0	1	2	1	0.5	3.5	2	1	1	4	3	3	2.5	8.5	2.5	3	3	8.5	2	3	3.5	8.5
	M1	0	0	0	0	1	0	0	1	2	1.5	1	4.5	3	2	2.5	7.5	3.5	3	3	9.5	3	4	3	9.5	3	3.5	4	10.5
	M2	0	0	0	0	1	0	0	1	2	1.5	1	4.5	3	2	2.5	7.5	3	3	3	9	3	4	3	9	3	3.5	4	10.5
	M3	0	0	0	0	1	0	0	1	2	1.5	2	5.5	3	2	3	8	3.5	3	3.5	10	3	4	4	10	3	3.5	4	10.5
	M3	0	0	0	0	1.5	0	0	1.5	2.5	1.5	1.5	5.5	3	2	2	7	3	2.5	3	8.5	3.5	3	3	8.5	2	3.5	3.5	9
IgY treated Ps group	F1	0	0	0	0	1	0	1.5	2.5	2.5	1	1.5	5	2.5	1	2	5.5	4	2	3	9	4	2.5	3.5	9	3	3	4	10
	F2	0	0	0	0	1	0	1	2	3	1	1.5	5.5	3	1.5	2	6.5	4	2	3	9	4	3	3.5	9	3	3.5	4	10.5
	F3	0	0	0	0	1	0	1	2	2.5	0	1.5	4	3	1	2	6	4	2.5	3	9.5	4	3	3	9.5	3	3.5	3.5	10
	M1	0	0	0	0	1	0	1.5	2.5	1.5	1.5	2	5	2	2	2.5	6.5	3	2.5	3	8.5	3.5	3	3.5	8.5	3	3.5	4	10.5
	M2	0	0	0	0	1	0	1	2	1.5	1	2	4.5	2	2	2.5	6.5	3	3	3.5	9.5	3.5	4	3.5	9.5	3	4	4	11
	M3	0	0	0	0	1	0	1.5	2.5	2	1.5	2	5.5	2.5	2	2.5	7	3	3	3.5	9.5	3.5	3.5	3.5	9.5	3	4	4	11
	F4	0	0	0	0	1.5	0	0	1.5	2.5	1	1	4.5	3	1.5	2	6.5	3.5	2	2.5	8	3.5	2.5	2.5	8	3	3	3	9
	F5	0	0	0	0	1.5	0	0	1.5	2	1	1	4	3	2	2	7	3.5	2.5	2.5	8.5	3.5	2.5	2.5	8.5	3	3	3	9
	F6	0	0	0	0	1.5	0	0	1.5	2	1	1	4	2.5	1.5	1.5	5.5	3.5	2.5	2.5	8.5	3.5	2.5	2.5	8.5	3	3	3	9
	M4	0	0	0	0	1	0	0	1	2	1	1	4	3	1.5	1.5	6	3	3	2.5	8.5	3	3	3.5	8.5	2.5	3.5	3.5	9.5
	M5	0	0	0	0	1.5	0	0	1.5	2.5	1	1	4.5	3	1.5	1.5	6	3.5	2	2.5	8	3.5	2.5	3	8	3	3.5	3.5	10
	M6	0	0	0	0	1	0	0	1	2	1	1	4	2.5	1.5	1.5	5.5	3	2.5	2.5	8	3	2.5	3.5	8	2.5	3.5	3	9
	Average	0.0	0.0	0.0	0.0	1.1	0.0	0.4	1.5	2.1	1.1	1.2	4.4	2.6	1.6	2.0	6.2	3.3	2.6	2.8	8.7	3.4	3.2	3.3	8.7	2.8	3.5	3.7	9.9
	Stdev	0.0	0.0	0.0	0.0	0.2	0.0	0.6	0.6	0.4	0.3	0.4	0.6	0.4	0.4	0.4	0.8	0.4	0.4	0.4	0.6	0.4	0.5	0.4	0.6	0.4	0.3	0.4	0.7

Table S2. Individual histological parameters for each mouse from groups control, naturally remitted, psoriasis and IgY treated (- represents the absence of the investigated parameter, + low presence; ++ medium presence; +++ intense presence; ++++ extremely high presence)

Parameter	Control group		Naturally remitted		Psoriasis		IgY treated	
	Mouse nb.	Presence	Mouse nb	Presence	Mouse nb	Presence	Mouse nb	Presence
Vessels dilated and tortuous	1	-	1	-	1	++	1	+
	2	-	2	-	2	+++	2	-
	3	-	3	+	3	+++	3	-
	4	-	4	-	4	++++	4	+
	5	-	5	-	5	++	5	-
	6	-	6	-	6	+++	6	-
	7	-	7	-	7	+++	7	-
	8	-	8	-	8	+++	8	-
							9	-
							10	+
							11	-
							12	-
0edema and lymphocytic infiltrate	1	-	1	-	1	+++	1	-
	2	-	2	-	2	+++	2	+
	3	-	3	+	3	+++	3	-
	4	-	4	-	4	++++	4	-
	5	-	5	-	5	+++	5	-
	6	-	6	-	6	+++	6	-
	7	-	7	-	7	+++	7	-
	8	-	8	-	8	+++	8	-
							9	-
							10	+
							11	-
							12	-
Acanthosis	1	-	1	-	1	++++	1	-
	2	-	2	-	2	++++	2	-
	3	-	3	-	3	++++	3	-
	4	-	4	-	4	++++	4	-
	5	-	5	-	5	++++	5	-
	6	-	6	-	6	++++	6	-

	7	-	7	-	7	++++	7	-
	8	-	8	-	8	++++	8	-
							9	-
							10	-
							11	+
							12	-
Hyperkeratosis	1	-	1	-	1	++++	1	-
	2	-	2	-	2	++++	2	-
	3	-	3	-	3	++++	3	-
	4	-	4	+	4	++++	4	-
	5	-	5	-	5	++++	5	-
	6	-	6	-	6	++++	6	-
	7	-	7	-	7	++++	7	-
	8	-	8	-	8	++++	8	-
							9	-
							10	+
							11	-
							12	-
Parakeratosis	1	-	1	-	1	+++	1	-
	2	-	2	-	2	+++	2	+
	3	-	3	-	3	+++	3	-
	4	-	4	-	4	+++	4	-
	5	-	5	-	5	++	5	-
	6	-	6	-	6	+++	6	-
	7	-	7	-	7	+++	7	-
	8	-	8	-	8	+++	8	-
							9	-
							10	+
							11	+
							12	-
Elongation of the rete ridges	1	-	1	-	1	++++	1	-
	2	-	2	-	2	++++	2	-
	3	-	3	-	3	++++	3	-
	4	-	4	-	4	++++	4	-
	5	-	5	-	5	++++	5	-
	6	-	6	-	6	++++	6	-
	7	-	7	-	7	++++	7	-

	8	-	8	-	8	++++	8	-
							9	-
							10	-
							11	-
							12	-
